# PEA/Polydatin: Anti-Inflammatory and Antioxidant Approach to Counteract DNBS-Induced Colitis

**DOI:** 10.3390/antiox10030464

**Published:** 2021-03-16

**Authors:** Alessio Filippo Peritore, Ramona D’Amico, Marika Cordaro, Rosalba Siracusa, Roberta Fusco, Enrico Gugliandolo, Tiziana Genovese, Rosalia Crupi, Rosanna Di Paola, Salvatore Cuzzocrea, Daniela Impellizzeri

**Affiliations:** 1 Department of Chemical, Biological, Pharmaceutical, and Environmental Science, University of Messina, 98166 Messina, Italy; aperitore@unime.it (A.F.P.); rdamico@unime.it (R.D.); rsiracusa@unime.it (R.S.); rfusco@unime.it (R.F.); tgenovese@unime.it (T.G.); dimpellizzeri@unime.it (D.I.); 2 Department of Biomedical and Dental Sciences and Morphofunctional Imaging, University of Messina, 98166 Messina, Italy; cordarom@unime.it; 3 Department of Veterinary Science, University of Messina, 98166 Messina, Italy; egugliandolo@unime.it (E.G.); rcrupi@unime.it (R.C.); 4 Department of Pharmacological and Physiological Science, Saint Louis University School of Medicine, Saint Louis, MO 63104, USA

**Keywords:** IBD, palmitoylethanolamide, polydatin, oxidative stress, inflammation, SIRT1/NRF2, NF-κB

## Abstract

Palmitoylethanolamide (PEA) has well-known anti-inflammatory effects. However, PEA does not possess an antioxidant ability. A comicronized formulation of ultramicronized PEA (um-PEA) and polydatin (Pol) PEA/Pol, a biological precursor of resveratrol with antioxidant activity, could have protective effects on oxidative stress produced by inflammatory processes. We evaluated the effects of a comicronized PEA/Pol 10 mg/kg (9 mg of um-PEA+1 mg of polydatin) in a model of Dinitrobenzene sulfonic acid (DNBS)-induced colitis. Ulcerative colitis was induced in mice by intrarectally injection of DNBS (4 mg in 100 µL of 50% ethanol per mouse). Macroscopic and histologic colon alterations and marked clinical signs were observed four days after DNBS and elevated cytokine production. The myeloperoxidase (MPO) activity assessed for neutrophil infiltration was associated with ICAM-1 and P-selectin adhesion controls in colons. Oxidative stress was detected with increased poly ADP-ribose polymerase (PARP) and nitrotyrosine positive staining and malondialdehyde (MDA) levels in inflamed colons. Macroscopic and histologic alterations minimized by oral PEA/Pol, as well as neutrophil infiltration, inflammatory cytokine release, MDA, nitrotyrosine, PARP and ICAM-1, and P-selectin expressions. The mechanism of action of PEA/Pol could be related to the sirtuin 1/nuclear factor erythroid 2-related factor 2 (SIRT-1/Nrf2) pathway and nuclear factor (NF)-κB. PEA/Pol administration inhibited NF-κB and increased SIRT-1/Nrf2 expressions. Our results show that PEA/Pol is capable of decreasing inflammatory bowel disease (IBD) DNBS-induced in mice.

## 1. Introduction

Inflammatory bowel disease (IBD) falls under the variety of severe inflammatory bowel disorders caused by the dysfunction of the intestinal epithelial barrier that leads to an increase in intestinal permeability [1]. IBD-included ulcerative colitis (UC) and ‘ ‘Crohn’s disease (CD) are characterized by the release of pro-inflammatory cytokines, for example, TNF-α (alpha tumor necrosis factor), causing further damage to the barrier function and perpetuating inflammation [2,3]. Indeed, in previous studies on mucosal biopsies of IBD patients, an increase in pro-inflammatory chemokines, cytokines, and adhesion molecule expression was observed [4]. Recently, several studies focused on reactive nitrogen and oxygen species (RNS and ROS) as etiologic elements for IBD [5]. The bowel is a principal place for the origination of pro-oxidants, whose formation is mainly due to an excess of food constituents, microbes, and communications between immune cells [5]. In addition, a decrease in antioxidant capacity was observed in patients with IBD and asymptomatic subjects [6]. To counteract RNS, intestinal cells require enzymatic and non-enzymatic antioxidants, for example, superoxide dismutase (SOD), but massive production of RNS provokes a lipid peroxidation (LP) increase that consequently can reduce antioxidant protection [7]. In some cases, the oxidative stress (OS) concomitant to immune activation and inflammation could contribute to tissue injury and fibrosis that characterize bowel diseases [8]. To date, the existing therapies for IBD include sulfasalazine; corticosteroids; immunosuppressive agents; and several biological drugs, for example, anti-TNF-α antibodies [9]. Nevertheless, these drugs have several adverse effects after long periods of treatment, and the risk of relapse limits their use [10]. Moreover, many patients with IBD show no clinical improvement with the current therapies [11]. Since OS and inflammation contribute to tissue damage during colitis, recently, the administration of antioxidants combined with further anti-inflammatory action as a treatment of IBD [12,13] was proposed. In the current study, we used a model of Dinitrobenzene sulfonic acid (DNBS)-induced colitis to evaluate the effects of a PEA/Pol compound on the regulation of inflammatory processes and oxidative stress. PEA is an endogenous lipid contained in several foods that belongs to the ALIAmides (autocoid local injury antagonism) family [14,15]. PEA has important anti-inflammatory and analgesic properties on several molecular targets [16,17]. However, PEA lacks the direct antioxidant capacity to counteract damage to DNA, protein, and lipids, and at the same time, prevent the formation of free radicals. For this purpose, we evaluated the action of um-PEA comicronized (9 mg of um-PEA and 1 mg of polydatin) with the important antioxidant polydatin 3,4,5-trihydroxystylbene-3mono-DGlucoside, a natural glucoside of resveratrol. PEA/Pol has been proved to have numerous biological properties, such as protective anti-inflammatory and anti-oxidation effects on different organs [18].

## 2. Materials and Methods

### 2.1. Animals

CD1 male mice (25 g; Envigo, Milan, Italy) were housed in a well-ordered locality (room 22 ± 1 °C 12-h dark/light cycles) with ordinary rodent chow and water. The animals adjusted to these circumstances in one week. Messina University Review Board for animal care endorsed the research. All animal experiments agree with the new Italian regulations (D.Lgs 2014/26) and EU regulations (EU Directive 2010/63).

### 2.2. Colitis Induction and Drugs

The intrarectal injection of DNBS (4 mg in 100 µL of 50% ethanol per mouse) (Sigma-Aldrich, Milan, Italy) in mice was performed on day 0 for induction of colitis as previously reported; after four days, mice were sacrificed [19]. Epitech Group SpA provided the um-PEA, polydatin, and PEA/Pol (Saccolongo, Italy) for the study. We used sulforaphane (SF) as a positive control for the antioxidant effect of PEA/Pol.

### 2.3. Experimental Groups

Treatment administration was conducted orally for 4 days based on our previous studies on the effects of PEA on a mouse model of colitis induced by intrarectal DNBS injection [19,20].

Specifically, mice were divided into the following groups, with 6 mice for each group:

DNBS+vehicle: Mice were subjected to the above-described DNBS administration, and every 24 h, saline was administered orally for four days, beginning 1 h after DNBS.

DNBS+Pol: Mice were subjected to the above-described DNBS administration, and every 24 h, Pol 1 mg/kg was administered orally for four days, beginning 1 h after DNBS.

DNBS+um-PEA: Mice were subjected to the above-described DNBS administration, and every 24 h, um-PEA 9 mg/k was administered orally for four days, beginning 1 h after DNBS.

DNBS+PEA/Pol: Mice were subjected to the above-described DNBS administration, and every 24 h PEA/Pol 10 mg/kg, a comicronized compound with 9 mg of um-PEA and 1 of polydatin, was administered orally for four days, beginning 1 h after DNBS.

Sham-operated groups: vehicle solution (saline), Pol, um-PEA, and PEA/Pol (1, 9, and 10 mg/kg) were orally administered for four days. Since no significant histological or macroscopic change was found between the sham groups, we present data of sham+vehicle groups only for the other analysis.

To investigate the antioxidant pathway, a further positive control group was used with sulforaphane (SF), an activator of Nrf2, dissolved in salad oil at 20 mg/kg as previously described [21].

DNBS+SF: Mice were subjected to the above-described DNBS administration, and every 24 h, SF 20 mg/kg was administered orally for four days, beginning 1 h after DNBS.

### 2.4. Body Weight and Evaluation of Colon Damage

Animals were weighed every day, from day 0 until the day of sacrifice. The colon tissue was adequately removed and untied by a longitudinal incision. Two autonomous observers reported the degrees of gross damage [19].

### 2.5. Histological Examination

Tissues were treated with hematoxylin and eosin (H&E) staining for histological assessment; damage was semi-quantitatively scored from 0 to 4, as mentioned previously [18], in a blinded fashion by two qualified pathologists using a Leica DM6 microscope (Leica Microsystems SpA, Milan, Italy) associated with Leica LAS X Navigator software (Leica Microsystems SpA, Milan, Italy). The choice of photographing two different enlargements, 10× (for H&E) and 40x (for immunohistochemistry), was made to best highlight the results sought.

### 2.6. Malondialdehyde (MDA) Assay

As previously reported, MDA levels were determined to detect lipid peroxidation four days after administration of DNBS in colon tissue [22,23].

### 2.7. Myeloperoxidase Activity

Myeloperoxidase (MPO) activity, an indicator of PMN accumulation, was determined spectrophotometrically at 650 nm [24,25,26]. MPO activity was expressed in unit per gram of wet tissue weight and was measured as the quantity of enzyme degrading 1 mM of peroxide min^−1^ at 37 °C.

### 2.8. Immunohistochemical Localization of Cell Adhesion Molecules (ICAM-1, P-Selectin), Poly (ADP-Ribose Polymerase) (PARP), and Nitrotyrosine

Immunohistochemical analysis was performed as previously described [27,28], 4 days after DNBS administration. The sections were incubated overnight with primary antibodies anti-ICAM-1 mouse polyclonal antibody (1:100 in Phosphate-buffered saline (PBS), *v*/*v*, Santa Cruz Biotechnology SCB, D.B.A, Milan, Italy), anti-P-selectin mouse polyclonal antibody (1:100 in PBS, *v*/*v*, SCB, D.B.A, Milan, Italy), anti-PARP mouse polyclonal antibody (1:100 in PBS, *v*/*v*, SCB, D.B.A, Milan, Italy), and anti-nitrotyrosine rabbit polyclonal antibody (1:200 in PBS, v/v, Millipore, D.B.A, Milan, Italy). All sections were washed with PBS and then treated as previously reported [29,30]. Five stained sections from each mouse were scored in a blinded fashion and observed using a Leica DM6 microscope (Leica Microsystems SpA, Milan, Italy) following a typical procedure [31]. The histogram profile is related to the positive pixel intensity value obtained [32].

### 2.9. Western Blots Analyses

Cytosolic and nuclear extracts were prepared as previously described on colon tissues [25,33]. The following primary antibodies were used: anti-IκB-α (SCB, 1:500 #sc1643, D.B.A, Milan, Italy), anti-NF-κB p65 (SCB; 1:500 #sc8008), anti-HO-1 (1:1000; StressGen Biotech), anti-iNOS (BD Transduction Laboratories, 1:500), anti-MnSOD (Millipore, 1:500, Cat 06-984, D.B.A, Milan, Italy), anti-Nrf2 63kDa (1:500; A-10:sc-365949; Santa Cruz Biotechnology), and anti-SIRT1 (1:500, Santa Cruz Biotechnology) in PBS (Biogenerica srl, Catania, Italy), 5% *w/v* non-fat dried milk, 0.1% Tween-20 at 4 °C overnight. Membranes were incubated with peroxidase-conjugated bovine anti-mouse IgG secondary antibody or peroxidase-conjugated goat anti-rabbit IgG (Jackson ImmunoResearch, West Grove, PA, USA; 1:2000) for 1h at room temperature. Anti-β-actin or anti-lamin A/C (D.B.A, Milan, Italy) antibodies were used as controls. Protein expression was analyzed as previously reported [34].

### 2.10. Cytokine Measurements

As previously described [35,36], in the colon tissues obtained after sacrifice, TNF-α (Ray Bio ELISA Kit MouseTNF-alpha, Norcross, GA, USA) and interleukin IL-1β ((R&D Systems, Milan, Italy) levels were evaluated using a colorimetric commercial kit.

### 2.11. Statistical Evaluation

All values are stated as mean ± standard error of the mean (SEM) of N observations. N represents the number of animals. For histology and immunohistochemistry, the photographs are the outcomes of at least three independent experimentations. A *p*-value of less than 0.05 was significant. One- or two-way ANOVA followed by a Bonferroni post hoc test for multiple comparisons were used.

## 3. Results

### 3.1. Effects of PEA/Pol on Macroscopic Change and Body Weight

**Figure 1:**
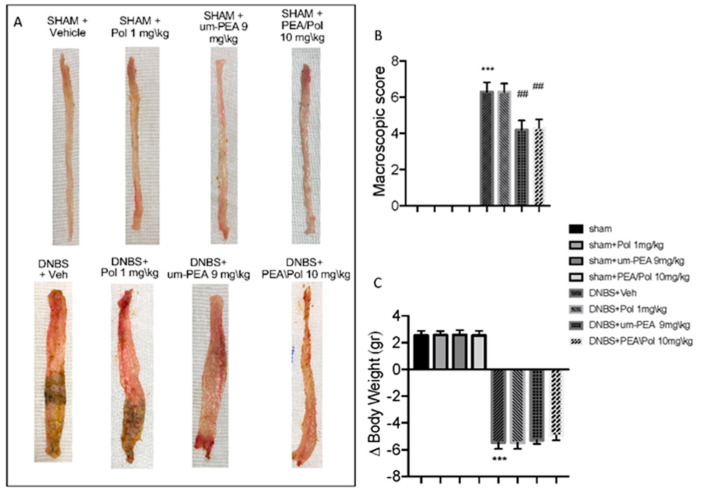
The effects of palmitoylethanolamide/polydatin (PEA/Pol) on macroscopic damage and body weight after four days of Dinitrobenzene sulfonic acid (DNBS)-injection. Macroscopic damage in sham, sham+Pol, sham+ultramicronized PEA (um-PEA), sham+PEA/Pol, DNBS+vehicle (Veh), DNBS+Pol, DNBS+um-PEA, and DNBS+PEA/Pol groups (**A**). Macroscopic score (**B**). Body weight (**C**). Values = means ± SEM of six animals in each group; *** *p* < 0.001 vs. sham; ^##^ *p* < 0.01 vs. DNBS.

No macroscopic change was observed in the colon from sham animals. On day 4, animals treated with DNBS presented a colon flaccid and filled with liquid stool and, in some cases, ulcerative damages with mucosal congestion (Figure 1A). Oral PEA/Pol therapy at 10 mg/kg and above um-PEA alone at 9 mg/kg decreased inflammation in the DNBS-injected mouse compared to vehicle (Figure 1A). Moreover, all mice showed a decrease in body weight compared to sham groups. PEA/Pol administration ameliorated clinical symptoms of colon inflammation and body weight loss. (Figure 1A–C).

### 3.2. Effects of PEA/Pol on Histological Colon Damage

**Figure 2:**
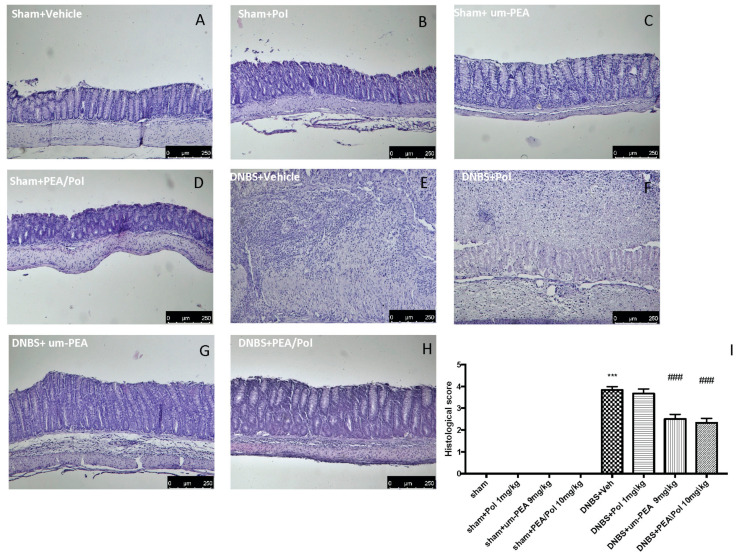
The effects of PEA/Pol on histological damage after DNBS injection. Histological analysis was evaluated in sham (**A**), sham+Pol (**B**), sham+um-PEA (**C**), sham+PEA/Pol (**D**), DNBS+Veh (**E**), DNBS+Pol (**F**), DNBS+um-PEA (**G**), and DNBS+PEA/Pol (**H**). The histological score was measured (**I**). Images are figurative of at least three independent experiments. Values = means ± SEM of six animals in each group; *** *p* < 0.001 vs. sham; ^###^ *p* < 0.001 vs. DNBS.

No histological modification was observed in the colon tissue of sham mice (Figure 2A–D). Clear leukocyte infiltration, necrosis, and edema were observed in the sections of the colon of DNBS-injected mice (Figure 2E). The oral treatment with PEA/Pol 10 mg/kg reduced histological impairments more than um-PEA alone at 9 mg/kg (Figure 2G,H) did. Treatment of single Pol at a dose of 1 mg/kg showed no effect on histological impairment after DNBS injection (Figure 2F).

### 3.3. Effects of PEA/Pol on Neutrophil Infiltration, Cytokine Levels, and Lipid Peroxidation

**Figure 3:**
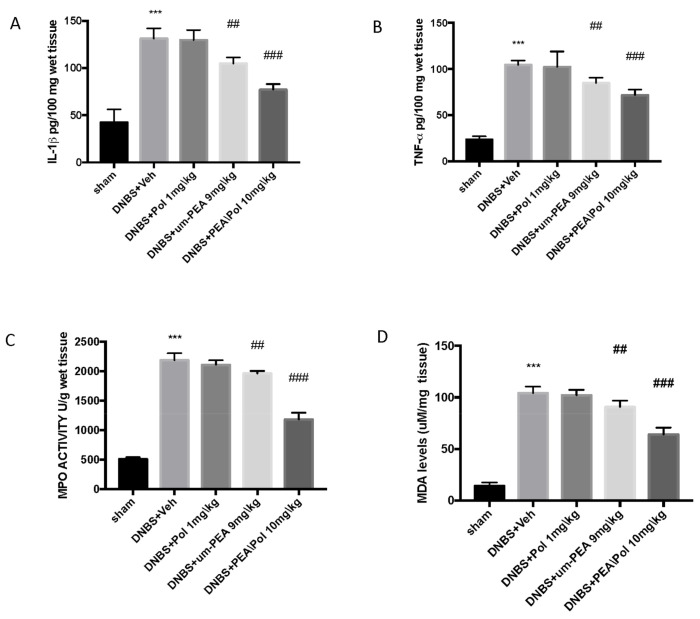
The effects of PEA/Pol on cytokine, myeloperoxidase (MPO) activity, and malondialdehyde (MDA) levels. IL-1β (**A**), TNF-α (**B**), MPO (**C**), and MDA (**D**) levels were examined. PEA/Pol treatment reduces cytokines, MPO activity, and MDA levels. Values = means ± SEM of six animals in each group; *** *p* < 0.001 vs. sham; ^##^ *p* < 0.01 vs. DNBS. ^###^ *p* < 0.001 vs. DNBS.

Colon injury was also illustrated by an increase in MPO activity, an indicator of the neutrophil amassing in the colon. In addition, pro-inflammatory cytokine and lipid peroxidation levels triggered by neutrophil-derived superoxide anion were also observed to be increased in the MDA test. Mice exposed to DNBS demonstrated an increase in IL-1β and TNF-α release. (Figure 3A,B) as well as increased MPO activity (Figure 3C) and MDA levels (Figure 3D) when compared to sham groups.

PEA/Pol at 10 mg/kg, more than oral treatment of um-PEA alone at 9 mg/kg, significantly reduced cytokine levels (such as IL-1β and TNF-α (Figure 3A,B) and MPO levels (Figure 3C) and caused a reduction in MDA (Figure 3D).

### 3.4. Effects of PEA/Pol on Nitrotyrosine and PARP Expression

**Figure 4:**
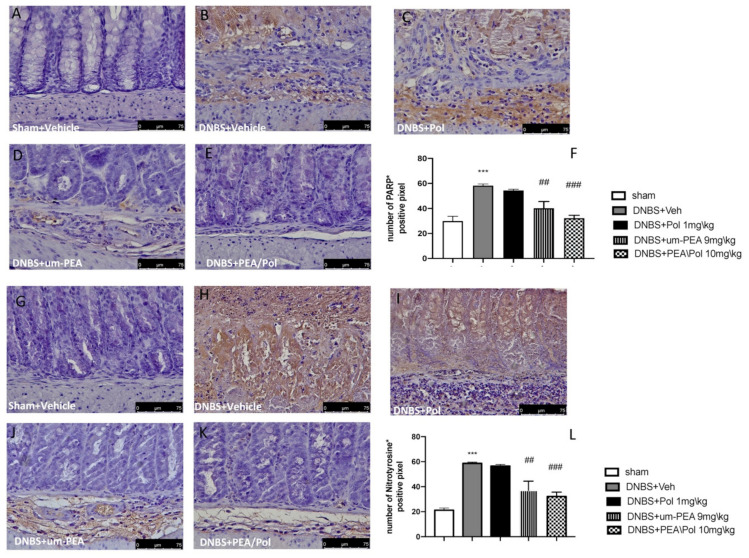
The effects of PEA/Pol on PARP and nitrotyrosine expression after DNBS injection. Immunohistochemistry for PARP was evaluated in sham (**A**), DNBS+Veh (**B**), DNBS+Pol (**C**), DNBS+um-PEA (**D**), and DNBS+PEA/Pol (**E**). The results are expressed as % of positive pixels (**F**). Images are figurative of at least three independent experiments. Immunohistochemistry for nitrotyrosine was evaluated in sham (**G**), DNBS+Veh (**H**), DNBS+Pol (**I**), DNBS+um-PEA (**J**), and DNBS+PEA/Pol (**K**). The results are expressed as % of positive pixels (**L**). Images are figurative of at least three independent experiments. Values = means ± SEM of six animals in each group; *** *p* < 0.001 vs. sham; ^##^ *p* < 0.01 vs. DNBS, ^###^ *p* < 0.001 vs. DNBS.

Colon sections from sham mice (Figure 4A,G) did not show nitrotyrosine expression, while the colon of DNBS-treated mice showed a robust, favorable stain (Figure 4G,H). Moreover, we observed a more positive stain of PARP in the colon tissues of the DNBS-injected mouse compared to sham groups. (Figure 4A,B). Um-PEA alone administration at 9 mg/kg reduced positive staining for nitrotyrosine and PARP, while an effect was not observed for Pol 1 mg/kg alone (Figure 4C–E,I–K). However, PEA/Pol at 10 mg/kg shows a more significantly positive effect than um-PEA alone (Figure 4E,K). Figure 4F,L show the number of positive stains for nitrotyrosine and PARP, where both um-PEA and PEA/Pol show a significant decrease compared to DNBS groups. Among the two, PEA/Pol shows a more significant decrease than um-PEA alone.

### 3.5. Effects of PEA/Pol on ICAM-1 and P-Selectin Expression

**Figure 5:**
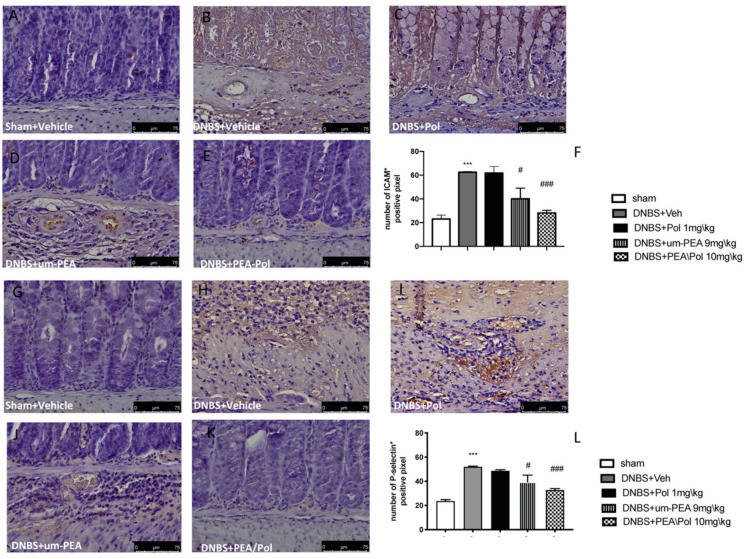
The effects of PEA/Pol on P-selectin and ICAM-1 expression after DNBS injection. Immunohistochemistry for ICAM-1 was evaluated in sham (**A**), DNBS+Veh (**B**), DNBS+Pol (**C**), DNBS+um-PEA (**D**), and DNBS+PEA/Pol (**E**). The results are expressed as % of positive pixels (**F**). Images are figurative of at least three independent experiments. Immunohistochemistry for P-selectin was evaluated in sham (**G**), DNBS+Veh (**H**), DNBS+Pol (**I**), DNBS+um-PEA (**J**), and DNBS+PEA/Pol (**K**). The results are expressed as % of positive pixels (**L**). Images are figurative of at least three independent experiments. Values = means ± SEM of six animals in each group; *** *p* < 0.001 vs. sham; ^#^ *p* < 0.01 vs. DNBS, ^###^ *p* < 0.001 vs. DNBS.

Intestinal expression of ICAM-1 and P-selectin are also implicated during cell enrollment. No positive staining was found in sham mice (Figure 5A,G). Positive staining for ICAM-1 (Figure 5B) and P-selectin (Figure 5H) was prominently increased in tissues from DNBS-injected mice. The oral treatment with um-PEA 9 mg/kg was able to reduce the positive staining for ICAM-1 and P-selectin (Figure 5D,J); conversely, Pol 1 mg/kg did not show a positive effect (Figure 5C,I). PEA/Pol 10 mg/kg shows a better result compared to um-PEA alone (Figure 5E,K). In Figure 5F,L, the number of positive staining for ICAM and P-selectin shows a decrease compared to the DNBS groups for both PEA/Pol and um-PEA treatments, where PEA/Pol presents a more significant action.

### 3.6. Effects of PEA/Pol on Inflammatory Pathway and Nitrosative Stress

**Figure 6:**
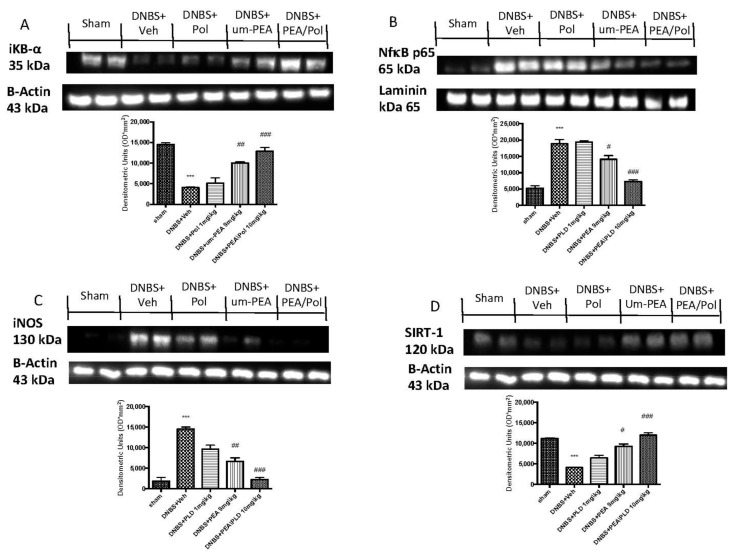
Western blots for NF-kB, IKB-α, iNOS, and SIRT-1. Representative Western blots for cytoplasmic IKB-α degradation (**A**), nuclear NF-κB translocation (**B**), cytoplasmic iNOS (**C**), and cytoplasmic SIRT-1 expression (**D**) were performed. A demonstrative blot of lysates (six animals/group), with a densitometric analysis for all animals, is shown. The results in (**A**–**D**) = means ± SEM of six animals in each group. *** *p* < 0.01 vs. sham, ^#^ *p* < 0.05 vs. DNBS., ^##^ *p* < 0.01 vs. DNBS, ^###^ *p* < 0.001 vs. DNBS.

Western blots for the NF-κB pathway were performed to investigate the beneficial effects of PEA/Pol in the inflammatory process. On day 4, DNBS-injected mice showed an increase in NF-κB p65 levels compared to animals without treatment (Figure 6A). In addition, IκB-α levels were decreased in DNBS+Veh mice, while sham groups showed homeostatic expression of IκB-α (Figure 6B). PEA/Pol treatment, more than PEA administration alone, reduced nuclear translocation of NF-κB p65 and, at the same time, IKB-α degradation (Figure 6A,B). In order to study the effects of PEA/Pol on nitrosative stress, we examined the expression of nitric oxide (NO) by evaluation of pro-inflammatory enzyme iNOS expression, commonly related to NF-κB activation (Figure 6C). DNBS injection resulted in increased iNOS expression (Figure 6C). Oral PEA/Pol administration could reduce iNOS more than um-PEA alone. (Figure 6C). Western blots for SIRT-1 were performed to further investigate the pathway in the beneficial effects of PEA/Pol. SIRT-1 levels decreased four days after DNBS injection when compared to sham groups (Figure 6D). The treatment with PEA/Pol, more than um-PEA treatment alone, was able to increase SIRT-1 expression (Figure 6D).

### 3.7. Effects of PEA/Pol on Nrf2 Pathway and Oxidative Stress

**Figure 7:**
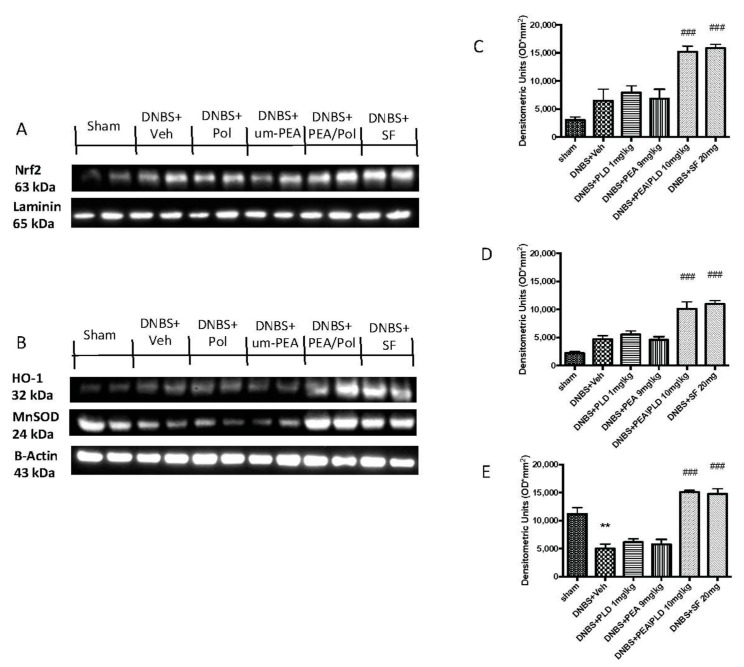
Western blots for Nrf2, HO-1, and manganese superoxide dismutase (MnSOD). Representative Western blots for nuclear Nrf2 translocation (**A**), cytoplasmic HO-1 expression, and cytoplasmic MnSOD expression (**B**) were performed. Densitometric analysis for Nrf2 (**A**), HO-1 (**B**), and MnSOD (**C**) are shown. The results in (**C**–**E**) = means ± SEM of six animals in each group; ** *p* < 0.01 vs. sham, ^###^ *p* < 0.001 vs. DNBS.

To examine the antioxidative action of PEA/Pol, we investigated Nrf2 accumulation and HO-1 induction, which are indicative of Nrf2 activation. We evaluated the expression of Nrf2 with a molecular weight of 63 kDa, as indicated to the company, although several studies have shown that the molecular weight of Nrf2 could be greater, approximately between 90 and 110 kDa [37,38]. Moreover, we compared the action of PEA/Pol to that of a positive control SF, commonly known to possess a strong ability to induce the transcription of Nrf2. DNBS injection caused a non-significant increase in expression of HO-1 as well as an Nrf2 nuclear expression (Figure 7A,B). The oral treatment with PEA/Pol, as well as SF, was able to increase HO-1 and Nrf2 expression four days after DNBS, while we did not observe the same action for um-PEA and polydatin alone (Figure 7A,B).

Furthermore, we analyzed the antioxidant effects of PEA/Pol on manganese superoxide dismutase (MnSOD) levels (Figure 7C). DNBS injection resulted in reduced antioxidant protection with decreased MnSOD expression (Figure 7C). Oral PEA/Pol administration, in the same way as SF, significantly increased MnSOD expression, contrary to um-PEA and polydatin treatment alone. (Figure 7C).

## 4. Discussion

In the present study, we investigated the antioxidant and anti-inflammatory properties of PEA/Pol. The results reveal that DNBS-induced colon inflammation in the mouse could be prevented by PEA/Pol, demonstrating its ability to manage IBD in mice though the effects of two single compounds. At the macroscopic and histologic levels, epithelial disruption was significantly reduced in mice treated with PEA/Pol. Furthermore, mice treated with PEA/Pol showed a better response to DNBS-induced colitis, presenting a wide resolution of the inflammatory process in terms of edema, infiltration of neutrophils, and ulcer formation. This was also in line with bodyweight data that showed a decrease in weight loss and MPO activity reduction after PEA/Pol treatment compared with the DNBS-injected group. NF-κB is an important mediator of inflammation, and its activation can be induced by stimuli, such as lipopolysaccharides, pro-inflammatory cytokines, and DNA damaging agents [39]. In response to these wide-ranging stimuli, including infection, oxidative stress, extracellular signals, hypoxia, and inflammation, IkBα is phosphorylated by IkB kinase [40].

DNBS induced a prominent degradation of IkBα and consequently an increase in the p65 nuclear translocation, a subunit of NF-κB, whereas PEA/Pol treatment significantly reduced NF-κB translocation and inhibited IkBα degradation.

IBD is characterized by an increase in several cytokines, such as IL-1β and TNF-α, but also prostaglandins and NO, which could damage barrier function and muscle contraction [41].

Intracellular signal transduction, such as the NF-κB pathway, controls the release of IL-1 β and TNF-α pro-inflammatory cytokines. After activation, NF-κB also influences the survival and proliferation of cells, adhesive molecules (ICAM, for example), and the expressions of growth factors that affect the progression of intestinal inflammation.

During inflammation in the bowel, several cells and macrophages can produce wide quantities of TNF-α. In endothelial cells, TNF-α overproduction can intensify ICAM expression, an important adhesion molecule, thereby significantly stimulating leukocyte infiltration into the intestinal mucosa. In the current study, PEA/Pol also resulted in a decrease in the TNF-α and IL-1β cytokines, which are related to the NF-κB pathway, but also P-selectin and ICAM-1 levels that can be caused by increased gut injury. Our results are in line with a previous study in which the oral treatment with PEA/Pol in the model of vascular injury reduced the inflammatory process by decreasing adhesion molecules, neutrophil infiltration, as well as pro-inflammatory cytokine production and NF-κB translocation [18]. Chronic intestinal inflammation has been recognized as a result of oxidative and nitrosative stress [42], which are involved in various human diseases, including IBD. A considerable amount of evidence suggests that IBD is connected with a discrepancy between antioxidant activity and ROS that generates oxidative stress as a consequence of this antioxidant activity decrease or ROS overproduction. An increase in ROS levels has damaging effects that can affect proteins, nucleic acids, and lipids through instigating fragmentation products causing enzymatic alteration, lipid peroxides development, and DNA strand break products [43].

Moreover, in the pathogenesis of bowel inflammation, iNOS play an important role in increasing NO levels that lead to a peroxynitrite over-production and consequently provoke injurious alterations in protein structure and functionality [44].

PEA is an anti-inflammatory compound that has shown its therapeutic effects on several experimental models, such as colitis, vascular lesions, skin wounds, neuropathy, and many others, but it appears to lack antioxidant action [40,45,46]. In the present study, we have shown that the addition of polydatin to um-PEA in a comicronized formulation gave um-PEA an antioxidant capacity that allows it to counteract oxidative stress and ROS formation by decreasing iNOS expression, PARP, and nitrotyrosine levels, as well as increasing MnSOD levels, an antioxidative enzyme involved in oxidative stress.

To better understand the mechanisms of PEA/Pol, we studied an important antioxidative pathway, SIRT1/Nrf2, that seems to be correlated with NF-κB signaling. A previous study showed that the SIRT1/Nrf2 signaling pathway can play a key role in alleviating oxidative stress and inflammation [47]. Several studies have shown the involvement of SIRT1 in the regulation of some physiological processes as well as oxidative stress and inflammation [48,49], and Nrf2 is the major transcriptional regulator of antioxidant proteins that translocates to the nucleus and promotes the expression of HO after cell injury [50].

It is known that increased levels of ROS induce the activation of Nrf2 and NF-κB [51]. Therefore, our hypothesis was that PEA/Pol could act on Nrf2 by keeping the antioxidant pathway active and inhibiting ROS release by negative feedback. Indeed, from the results obtained, we have shown that DNBS-induced depletion of SIRT1 could decrease the binding activity of the transcription factor Nrf2 in the nucleus, thereby reducing HO-1 expression.

PEA/Pol treatment significantly inhibited DNBS-induced downregulation of SIRT1, Nrf2, and HO-1, suggesting promotion of this pathway, as supported by the comparison with SF.

SF is one of the most abundant isothiocyanates in many cruciferous vegetables, particularly broccoli, which has shown in numerous studies a strong ability to activate the pathway of the antioxidant response element Nrf2 [52,53,54].

Both PEA/Pol and SF treatment showed an increase in Nrf2 antioxidant activity following DNBS-induced colitis as well as increased MnSOD expression levels, suggesting the involvement of the Nrf2 antioxidant pathway in the mechanism of protection of PEA/Pol compared to the action of um-PEA, which showed only anti-inflammatory effects.

At this point, given the connection between Nrf2 and NF-κB, we hypothesize that PEA/Pol could act with an anti-inflammatory effect on the NF-κB pathway, in line with previous studies [55,56,57], and also present an antioxidant activity by modulating Nrf2 and the other protein involved in its pathway.

## 5. Conclusions

In conclusion, our study has clearly established that the use of PEA/Pol, compared with um-PEA, presents some important differences that may clearly justify its use in chronic inflammatory disease with an oxidative stress component. Here, we showed the beneficial effects of PEA/Pol at doses of 10 mg/kg in a model of DNBS-induced colitis. The anti-inflammatory action of um-PEA, together with the antioxidative action of polydatin, could produce a protective action in the therapy for IBD. The PEA/Pol effect could be related to a decrease in inflammatory NF-κB activation signaling and an increase in the antioxidant SIRT1/Nrf2 pathway, which represent important targets of the pharmacological action against both inflammation and oxidative stress. Hence, this therapeutic strategy with the use of PEA/Pol could be important to combat ulcerative colitis in inflammatory bowel disease

## Data Availability

Data are included in the article.
