# Correction: Peritore et al. PEA/Polydatin: Anti-Inflammatory and Antioxidant Approach to Counteract DNBS-Induced Colitis. *Antioxidants* 2021, *10*, 464

**DOI:** 10.3390/antiox14040481

**Published:** 2025-04-17

**Authors:** Alessio Filippo Peritore, Ramona D’Amico, Marika Cordaro, Rosalba Siracusa, Roberta Fusco, Enrico Gugliandolo, Tiziana Genovese, Rosalia Crupi, Rosanna Di Paola, Salvatore Cuzzocrea, Daniela Impellizzeri

**Affiliations:** 1 Department of Chemical, Biological, Pharmaceutical, and Environmental Science, University of Messina, 98166 Messina, Italy; aperitore@unime.it (A.F.P.); rdamico@unime.it (R.D.); rsiracusa@unime.it (R.S.); rfusco@unime.it (R.F.); tgenovese@unime.it (T.G.); dimpellizzeri@unime.it (D.I.); 2 Department of Biomedical and Dental Sciences and Morphofunctional Imaging, University of Messina, 98166 Messina, Italy; cordarom@unime.it; 3 Department of Veterinary Science, University of Messina, 98166 Messina, Italy; egugliandolo@unime.it (E.G.); rcrupi@unime.it (R.C.); 4 Department of Pharmacological and Physiological Science, Saint Louis University School of Medicine, Saint Louis, MO 63104, USA

In the original publication [1], there was a mistake in Figure 1A as published. The corrected Figure 1 appears below.

The authors state that the scientific conclusions are unaffected. This correction was approved by the Academic Editor. The original publication has also been updated.

## Figures and Tables

**Figure 1:**
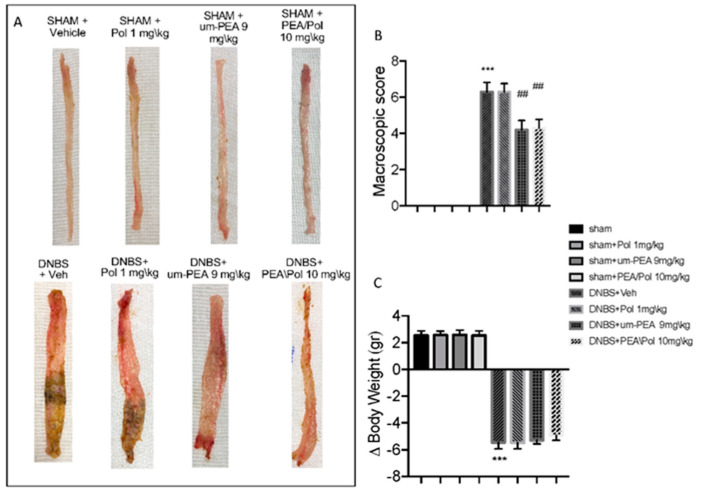
The effects of palmitoylethanolamide/polydatin (PEA/Pol) on macroscopic damage and body weight after four days of Dinitrobenzene sulfonic acid (DNBS)-injection. Macroscopic damage in sham, sham+Pol, sham+ultramicronized PEA (um-PEA), sham+PEA/Pol, DNBS+vehicle (Veh), DNBS+Pol, DNBS+um-PEA, and DNBS+PEA/Pol groups (**A**). Macroscopic score (**B**). Body weight (**C**). Values = means ± SEM of six animals in each group; *** *p* < 0.001 vs. sham; ^##^ *p* < 0.01 vs. DNBS.
